# Evaluation of human resources needed and comparison with human resources available to implement emergency vaccination in case of foot and mouth disease outbreaks in Tunisia

**DOI:** 10.1017/S0950268820001284

**Published:** 2020-06-18

**Authors:** Maud Marsot, Benoit Durand, Wafa Ben Hammouda, Heni Hadj Ammar, Malek Zrelli, Roukaya Khorchani

**Affiliations:** 1 University Paris Est, ANSES, Laboratory for Animal Health, Epidemiology Unit, Maisons-Alfort, France; 2 Tunisian Veterinary Services, 30 rue Alain Savary, 1002 Tunis, Tunisia

**Keywords:** FMD outbreak, human resources, Tunisia, vaccination

## Abstract

Foot and mouth disease (FMD) is a highly contagious viral disease that affects domestic and wild artiodactyl animals and causes considerable economic losses related to outbreak management, production losses and trade impacts. In Tunisia, the last FMD outbreak took place in 2018–2019. The effectiveness of control measures implemented to control FMD depends, in particular, on the human resources used to implement them. Tunisia has the ultimate objective of obtaining OIE status as ‘FMD-free with vaccination’. The aim of this study was to determine and compare the necessary and available human resources to control FMD outbreaks in Tunisia using emergency vaccination and to assess the gaps that would play a role in the implementation of the strategy. We developed a resources-requirement grid of necessary human resources for the management of the emergency vaccination campaign launched after the identification of a FMD-infected premises in Tunisia. Field surveys, conducted in the 24 governorates of Tunisia, allowed quantifying the available human resources for several categories of skills considered in the resources-requirement grid. For each governorate, we then compared available and necessary human resources to implement vaccination according to eight scenarios mixing generalised or cattle-targeted vaccination and different levels of human resources. The resources-requirement grid included 11 tasks in three groups: management of FMD-infected premises, organisational tasks and vaccination implementation. The available human resources for vaccination-related tasks included veterinarians and technicians from the public sector and appointed private veterinarians. The comparison of available and necessary human resources showed vaccination-related tasks to be the most time-consuming in terms of managing a FMD outbreak. Increasing the available human resources using appointed private veterinarians allowed performing the emergency vaccination of animals in the governorate in due time, especially if vaccination was targeted on cattle. The overall approach was validated by comparing the predicted and observed durations of a vaccination campaign conducted under the same conditions as during the 2014 Tunisian outbreak. This study could provide support to the Tunisian Veterinary Services or to other countries to optimise the management of a FMD outbreak.

## Introduction

Foot and mouth disease (FMD) is a highly contagious viral disease that affects domestic and wild artiodactyl animals, especially cattle, sheep, goats and pigs. FMD causes considerable economic losses, including production-related, costs for outbreak management, as well as indirect losses [1]. Although FMD has been eradicated in many countries, the disease is still largely present on the African and Asian continents. Despite strict preventive measures (strict control of the formal trade of animals and their products, increased surveillance, particularly for illegal transport, etc.), the virus can be introduced into a free country *via* infected animals or animal products [2, 3]. The measures to control FMD outbreaks include, among others, depopulation of infected herds and establishing a restriction zone around them, in which movement is restricted and herd surveillance performed. The purpose of clinical surveillance of herds within the restriction zone is to detect infected herds early and thus limit the spread of the disease [4]. Additional control measures, such as emergency vaccination and/or preventive depopulation [5], may be considered to control an expanding or already widespread outbreak.

In Tunisia, livestock farming, mainly small-scale production of cattle or small ruminants, is an important component of agricultural production, contributing to food security and alleviating poverty. The Veterinary Services in Tunisia consist of a central administration, the General Directorate of Veterinary Services, which is one of the nine General Directorates of the Ministry of Agriculture. The territorial services are Animal Production districts, which are part of the 24 Regional Agricultural Development Commissions (RADC). These official veterinary services collaborate with a network of appointed private veterinarians (*n* = 260 in 2020) to carry out certain veterinary prophylaxis. Tunisia has experienced several episodes of FMD outbreaks since 1975. In 2014, 150 FMD-infected premises of the serotype O/ME-SA/Ind-2001 were reported throughout Tunisia over 20 of the 24 governorates [6]. Specific measures were taken as soon as the first FMD-infected premises was confirmed to prevent the spread of the disease, mainly increasing vaccination coverage for cattle and small ruminants [7]. In 2017, two FMD-infected premises with the serotype A/AFRICA/G-IV were identified in Tunisia. The last outbreak in Tunisia took place from December 2018 to April 2019, with 14 FMD-infected premises (serotype O/EA-3) distributed among five governorates. As a member of the Mediterranean Animal Health Epidemiological Surveillance Network (REMESA), Tunisia has the specific objective to implement efficient and achievable control measures against FMD. However, these last FMD experiences revealed many difficulties in the management of FMD outbreaks, especially in terms of limited human resources to implement control measures.

The effectiveness of control measures depends on the resources used to implement them [8]. The management of the outbreak in Great Britain in 2001 identified human resources to be a limiting factor in determining the control measures chosen [9]. Human resources were also a major constraint during the management of the 2001 FMD outbreak in the Netherlands [10]. Surveillance in restriction zones and detection of new outbreaks appear to be crucial to minimise losses caused by the spread of the disease from the primary FMD-infected premises. However, this surveillance depends on the monitoring capacity (number of herds monitored per day), i.e. on the human resources available to implement such an operation. For example, during the 2014 outbreak in Tunisia, clinical surveillance could not be carried out, mainly due to the lack of human resources. Studies have shown that a reduction in surveillance capacity, closely linked to human resources, can lead to an increase in the duration of the outbreak, as well as to a larger number of infected herds and additional economic losses [4].

Human resources were identified as a major factor in the management of an FMD outbreak, both in terms of time and cost. Tunisia, along with its North African neighbours, is embarking on a disease-control strategy, with the ultimate objective of obtaining OIE status as ‘FMD-free with vaccination’. The primary objective of this study was to estimate the available and necessary human resources to control a FMD outbreak in Tunisia using emergency vaccination and to assess the gaps if any between required and available resources, in order to adjust to the situation before a crisis occurs. From a regulatory point of view, only veterinarians from the public sector are requisitioned in Tunisia to carry out emergency vaccination tasks. The second aim of this study was to test whether the inclusion of appointed private veterinarians could be beneficial in the implementation of emergency vaccination in case of an FMD crisis.

## Materials and methods

### Human resources-requirement grid

A grid of necessary human resources for the management of one FMD-infected premises in Tunisia was developed by combining the various tasks provided for by Tunisia's national emergency response plan, with the authority necessary to perform these tasks [11]. As some of the measures included in the FMD control program of Tunisia (program validated by the OIE) could not be properly implemented during the FMD outbreak of 2014 (e.g. culling of affected animals in the infected premises, the prohibition of animal movements and clinical surveillance), we have considered only those tasks that were carried out in 2014:

- Investigation of suspect cases, laboratory diagnosis and contact tracing,
- Vaccination of all domestic ruminants in the affected governorates,
- Implementation of points of disinfection.

For each task, the time necessary (in hours) to complete the task and the amount of human resources per skill (veterinarian, technician, driver and administrative agent) were estimated. Depending on their nature, tasks were classified as herd level tasks (management of FMD-infected premises and vaccination) and organisational tasks (governorate level duties) (Table 1). The time needed to complete a herd-level task was estimated for the management of a single infected premises or for the vaccination of a single herd. The time needed to complete organisational tasks was estimated for a given day of the outbreak. Since human resources and time required to complete herd-level tasks (e.g. vaccination) depend on the type of herd, we considered three categories of herds: small or medium-sized herds with less than 50 cattle, large cattle herds with more than 50 animals and small ruminant herds. Furthermore, for each working day of a vaccination team, an average travel time of 2 h was added to the working time dedicated to the vaccination itself. The resulting grid was validated both by national experts and by the national veterinary authorities in Tunis.

**Table 1.** Resources-requirement grid giving for each task estimates of the duration (in hours) and number of personnel needed to fulfil the task by skill

Task group	Task	Duration (in hours)	Veterinarian	Technician	Driver	Administrative agent
FMD-infected premises management^a^	Detection	4	1	1	1	0
	Sample management	4	0	0	1	0
	Herd blocking	4	1	0	1	0
	Epidemiological survey	5	1	0	1	0
	Determination of road disinfection points	8	1	0	0	0
Organizational tasks^b^	Crisis unit	8	2	0	0	1
	Local communication	8	1	1	0	0
	Vaccination planning	8	1	0	0	1
Emergency vaccination^c^	Vaccination of a small bovine herd	0.75	1	1	1	0
	Vaccination of a large bovine herd	1	1	1	1	0
	Vaccination of a small ruminants herd	0.5	1	1	1	0

a Herd-level task, the needed human resources are estimated for the management of a single infected premises.

b Governorate-level task, the needed human resources are estimated for a single day of the outbreak.

c Herd-level task, the needed human resources are estimated for the vaccination of a single herd.

### Available human resources

Field surveys, consisting of interviews with animal health managers, were conducted in the 24 governorates of Tunisia to quantify the human resources locally available for each category of skill considered in the resources-requirement grid and to estimate the number of herds in the governorate (information needed to calculate the number of herds to be vaccinated per governorate). The participants were asked to quantify the following indicators:

- the number of veterinarians of public sector available in the governorate,
- the number of technicians of public sector available in the governorate,
- the number of administrative agents available in the governorate,
- the number of herds in the governorates per type (small or medium-sized herds with less than 50 cattle, large cattle herds with more than 50 animals and small ruminant herds).

The number of veterinarians of the private sector, which could be mobilised in each governorate for the management of FMD, was also estimated. We mainly met with the head of the animal production of the district in the governorate and the veterinarians in charge of animal health in the district. Depending on availability and necessity, other individuals may have been present, such as those associated with the management of FMD in animal production in the district, representatives of the National Guard and Civil Protection and private veterinarians.

### Total workload per task and skill

Using the resource-requirement grid, we first calculated the time required per type of human resources (expressed in number of hours) to carry out (i) the FMD-infected premises management tasks for single FMD-infected premises and (ii) the organisational tasks for a single day of the outbreak. The same calculation was done for each governorate, as it did not depend on the number of herds. The time required, per type of human resources, to complete an emergency vaccination campaign in each governorate, was also calculated by multiplying the number of herds in each category (small and medium cattle herds, large cattle herds and small ruminants herds) by the estimated time needed to vaccinate a single herd of each category. This total working time per skill was expressed in man-months.

### Time needed to complete the emergency vaccination

In Tunisia, the FMD control program provides for teams consisting of a veterinarian, a technician and a driver to carry out vaccination operations in the field. We divided the total working time needed to complete vaccination in each governorate by the number of such vaccination teams locally available, to obtain the time needed to complete the emergency vaccination campaign in the governorate. In case of a FMD crisis, we assumed that vaccination teams worked 8 hours a day, 6 days a week. In practice, targeted vaccination and optimising the management of human resources may allow to reduce the time needed to complete the vaccination campaign. In particular, the inclusion of appointed private veterinarians in the human resources used could be beneficial. Four distinct scenarios for the management of the human resources dedicated to vaccination were considered:

1. the standard level of public resources, with vaccination teams composed of an official veterinarian accompanied by a technician and a driver,
2. the maximum level of public resources, with vaccination teams either composed of an official veterinarian, or of a technician (this corresponds to how human resources were used during the 2014 FMD outbreak),
3. the combination of the standard level of public resources with private resources, vaccination teams being either public vaccination teams as in (1), or private vaccination teams composed of a private appointed veterinarian.
4. the combination of the maximum level of public resources with private resources, vaccination teams being either public vaccination teams as in (2), or private vaccination teams composed of a private appointed veterinarian.

These four scenarios were combined with two scenarios for the vaccinated population:

- generalised vaccination of all ruminants, or
- cattle-targeted vaccination

We thus analysed eight vaccination numbered (1A) to (4B). For each scenario, we computed the number of months needed to fulfil the emergency vaccination campaign in the governorate.

### Validation

During the Tunisian 2014 outbreak, the emergency vaccination campaign began with all cattle herds, as well as 1 million small ruminants (representing 20% of the country population). This first phase of the vaccination campaign reduced the number of outbreaks and was carried out in 4 months, using the maximum level of public health resources (scenario (2) above). It was followed by a second phase, in which all ruminant herds were vaccinated (those vaccinated in the 1^st^ phase of the vaccination campaign were thus vaccinated twice). During this second phase, vaccination was performed using the maximal level of the public resources, with the help of the appointed private veterinarians (scenario (4) above) in approximately 4 months. For each governorate, we predicted the time needed to complete both phases of the 2014 emergency vaccination campaign and we compared the resulting values with the observed durations in 2014.

## Results

### Human resources-requirement grid

After analysis of the national emergency response plan for FMD, we identified 11 tasks requiring the mobilisation of human resources for the management of a FMD outbreak from the time a FMD-infected premises is suspected in the country to the completion of the emergency vaccination campaign (Table 1):

Management of suspected and infected premises

- Detection, consisting of the mobilisation of a team composed of a veterinarian, a technician and a driver to carry out the clinical examination of the suspect herd, take samples and implement biosecurity measures for 4 hours for one herd.
- Sample management, consisting of sending the samples to the laboratory and their analysis for FMD. The samples are brought by a driver to the laboratory of the Tunisian Institute of Veterinary Research located in Tunis (4 hours per herd).
- Herd blocking, consisting of sequestering the herd, carried out by police officers, accompanied by one veterinarian and a driver, and taking 4 hours per herd,
- Epidemiological survey, consisting of surveying a herd by a veterinarian, accompanied by a driver and taking 5 hours for each herd surveyed. The veterinarian identifies the potential sources of FMD introduction in the herd (contact tracing),
- Determination and organisation of road disinfection points would require a veterinarian for 8 hours for each infected herd.

Organisational tasks at the governorate level

- Vaccination planning at the level of the governorate would require a full-time veterinarian. The vaccination planning consists in the census of herds in the governorate and count of animals present in each herd of the governorate,
- Implementation of a crisis unit by the governorate, consisting of coordinating and managing the crisis. The cell would require two full-time veterinarians and one full-time administrative agent.
- Local communication to the professionals and health authorities would require a veterinarian and a technician.

Implementation of vaccination in ruminant herds

- Vaccination consisting of the clinical examination and vaccination of animals in all herds of the governorate. For each vaccinated herd, as provided for by the Tunisian FMD emergency plan, the vaccination task would require a veterinarian, a driver and a technician. We considered that vaccinating a small or medium-sized bovine herd takes 45 min, a large bovine herd 1 hour and a small ruminant herd 30 min.

### Available human resources

Surveys in the 24 governorates allowed us to determine the available human resources per type (veterinarian, technician, administrative agent) that could be mobilised in the event of a FMD outbreak (Table 2). As several categories of human resources could perform the vaccination task (official veterinarians accompanied by their drivers, vaccination technicians, or even appointed private veterinarians), we also identified the number of these resources in each governorate (Table 2). For example, in the governorate of Ariana, the available human resources consisted of nine official veterinarians, four technicians, three vaccination technicians, three drivers, two administrative agents and two appointed private veterinarians (Table 2). This implied that the number of vaccination teams in scenarios 1A, 3A, 1B and 3B was limited to the number of drivers available.

**Table 2.** Available human resources in Tunisian governorates by skill

Governorate	Official veterinarian	Technician	Vaccination technician	Driver	Administrative agent	Appointed private veterinarian
Ariana	9	4	3	3	2	2
Beja	6	8	5	9	0	15
Ben Arous	6	5	3	7	5	4
Bizerte	13	7	6	6	4	15
Gabes	8	3	10	9	2	8
Gafsa	10	5	13	5	5	12
Jendouba	7	10	4	7	0	12
Kairouan	14	11	15	11	1	15
Kasserine	13	10	2	3	4	14
Kebili	8	8	5	5	2	3
Le Kef	11	14	7	10	11	10
Mahdia	16	11	1	9	2	15
Manouba	11	3	7	7	0	6
Medenine	11	3	0	12	2	11
Monastir	9	8	9	8	3	6
Nabeul	13	10	4	5	6	16
Sfax	14	8	8	13	5	15
Sidi Bouzid	10	16	8	8	3	18
Siliana	11	9	10	11	3	14
Sousse	10	6	5	4	4	13
Tataouine	8	2	6	8	1	4
Tozeur	7	2	16	5	4	4
Tunis	7	2	0	3	1	1
Zaghouan	6	0	8	7	1	12

### Total workload per task and skill

The total workload (expressed in number of working hours) per skill (veterinarian, technician, driver, or administrative agent) to implement the tasks related to the management of infected premises and the organisational tasks, were evaluated for one infected premises and 1 day of outbreak, respectively. We found that, for given infected premises, the outbreak management tasks would take 21, 4 and 17 h for the skills of veterinarian, technician and driver respectively. At the governorate level, the organisational tasks would represent, for each day of the outbreak, a total workload of 104, 32 and 40 h for veterinarians, technicians and administrative agents, respectively.

The time dedicated to vaccination in the governorates was identified as the major part of the workload. The predicted vaccination workload, expressed in working months (with one working month equal to 30.5 days of 8 h), is given in Table 3. According to the governorate, it ranged between 3 and 87 man-months for the generalised vaccination and varied between <1 month and 48 man-months for the cattle-targeted vaccination.

**Table 3.** Estimated total workload (in man-months) and duration (in months) of an emergency vaccination campaign in each governorate according to the scenario^a^

	Total workload (man-months)		Duration of the emergency vaccination campaign (months)								
Governorate	Generalised vaccination	Cattle-targeted vaccination	Scenario 1A	Scenario 2A	Scenario 3A	Scenario 4A^b^	Scenario 1B	Scenario 2B	Scenario 3B	Scenario 4B	2014 FMD outbreak^c^
Ariana	7	6	4	1	2	1	3	1	2	1	1
Beja	35	18	6	5	2	2	3	3	1	1	3
Ben Arous	7	4	2	1	1	1	1	1	1	1	1
Bizerte	52	36	15	5	4	3	10	3	3	2	4
Gabes	18	1	3	2	2	1	0	0	0	0	0
Gafsa	25	7	9	2	2	1	2	0	1	0	1
Jendouba	80	48	20	12	7	6	12	7	4	4	8
Kairouan	69	29	11	4	5	3	4	2	2	1	2
Kasserine	45	10	25	5	4	3	6	1	1	1	2
Kebili	18	0	6	2	4	2	0	0	0	0	0
Le Kef	43	19	7	4	4	3	3	2	2	1	2
Mahdia	49	27	9	5	4	3	5	3	2	1	3
Manouba	13	7	3	1	2	1	2	1	1	0	1
Medenine	55	4	8	8	4	4	1	1	0	0	2
Monastir	20	8	4	2	2	1	2	1	1	1	1
Nabeul	9	6	3	1	1	0	2	1	0	0	1
Sfax	31	5	4	2	2	1	1	0	0	0	1
Sidi Bouzid	87	46	18	8	6	4	10	4	3	2	5
Siliana	30	9	5	2	2	1	1	1	1	0	1
Sousse	25	9	11	3	3	2	4	1	1	1	1
Tataouine	10	0	2	1	1	1	0	0	0	0	0
Tozeur	9	1	3	1	2	1	0	0	0	0	0
Tunis	3	2	2	1	1	1	1	0	1	0	0
Zaghouan	16	6	4	2	1	1	1	1	0	0	1

a Scenarios are numbered *XY*: *X* = 1 denotes the standard use of public human resources, *X* = 2 the maximum level of public resources, *X* = 3 the standard use of public resources and of appointed private veterinarians, *X* = 4 the maximum level of public resources and of appointed private veterinarians; *Y* = A denotes the generalised vaccination and *Y* = B the cattle-targeted vaccination.

b Predicted duration of the 2^nd^ phase of the 2014 emergency vaccination campaign during which all the cattle and small ruminants herds were vaccinated, using the maximum level of available public resources and with the help of private appointed veterinarians.

c Predicted duration of the 1^st^ phase of the 2014 emergency vaccination campaign during which all the cattle herds and 20% of the small ruminants herds (1.2 million animals) were vaccinated, using the maximum level of available public resources, without the help of private appointed veterinarians.

### Time needed to complete the emergency vaccination

**Fig. 1.**
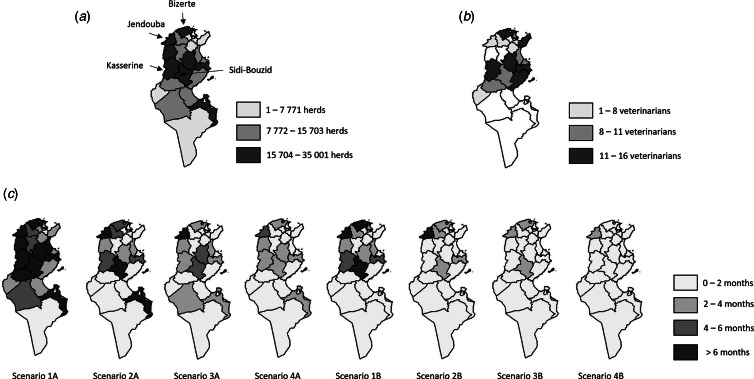
(a) Number of herds per governorate in Tunisia, (b) number of available veterinarians per governorate and (c) number of months needed for the vaccination of herds per governorate according to the scenario^1^ considered.

^1^Scenarios are numbered *XY*: *X* = 1 denotes the standard use of public human resources, *X* = 2 the maximum level of public resources, *X* = 3 the standard use of public resources and of appointed private veterinarians, *X* = 4 the maximum level of public resources and of appointed private veterinarians; *Y* = A denotes the generalised vaccination and *Y* = B the cattle-targeted vaccination.

The time needed to complete the emergency vaccination campaign in each governorate, given the available human resources, strongly varied according to the scenario (Table 3 and Fig. 1). Whatever the scenario, this duration was predicted to be notably longer in four governorates: Bizerte, Jendouba, Kasserine and Sidi Bouzid (Fig. 1). These governorates had a high density of cattle and small ruminants and Jendouba also had a low level of veterinarian resources (Fig. 1). The scenarios with cattle-targeted vaccination allowed completing the vaccination campaign in 6 months or less for scenario 1B (except for Bizerte, Jendouba and Sidi Bouzid) and in less than 4 months for the other scenarios (except for Jendouba in scenario 2B). The use of the standard level of public resources for vaccination was predicted to induce prolonged durations of emergency vaccination campaigns, especially for a generalised vaccination: under scenario 1A, the generalised vaccination campaign was predicted to last more than 6 months in 10 of24 governorates (Fig. 1 and Table 3). However, scenarios using appointed private veterinarians made it possible to limit the time dedicated to vaccination, and to complete the emergency vaccination campaign in 4 months or less in 23 of 24 governorates and in 2 months or less in 16 of 24 governorates. Finally, cattle-targeted vaccination using the maximum level of public resources and appointed private veterinarians (scenario 4B) was predicted to be completed in 4 months or less, irrespective of the governorate.

### Validation

The 1^st^ phase of the 2014 vaccination campaign, targeted on cattle herds and 20% of the small ruminant herds, using the maximum level of public resources, was predicted to last 4 months or less in all governorates except Jendouba (8 months) and Sidi Bouzid (5 months) (Table 3). The observed duration of 4 months was consistent. The 2^nd^ phase of the 2014 vaccination campaign was a generalised vaccination using the maximum level of public resources and appointed private veterinarians, thus corresponding to scenario 4A. It was predicted to last 4 months or less, except in Jendouba (6 months). The observed value of 4 months was consistent.

## Discussion

The management of FMD outbreaks depends on their extent and the human resources available to control the disease. In Tunisia, the control measures used during the 2014 outbreak included 11 tasks, from the detection of the first infected premises to the completion of the emergency vaccination campaign. We developed a resource-requirement grid in case of a FMD outbreak by defining the time necessary to fulfil these tasks (management of infected premises, organisational tasks and vaccination implementation) and the number of required associated human resources per category of skill (veterinarian, technician, or administrative agent). A field survey allowed us to evaluate the human resources available in each Tunisian governorate by skill. By combining these needed and available human resources, we have predicted how long it would take to complete an emergency vaccination campaign under the same conditions as during the Tunisian 2014 outbreak. The resulting durations were consistent with those observed in 2014, which validated our resources-requirement grid, at least for its part related to the emergency vaccination campaign (further studies are needed to validate the parts related to the management of infected premises and to the organisational tasks). These predicted durations were however somewhat longer in two of the 24 Tunisian governorates (Jendouba and Sidi Bouzid). In these governorates, in 2014, flocks were often brought by their owners to the assembly centres for vaccination especially for small ruminants, which allowed an optimisation of the vaccination tasks, although it increased the risk of FMD direct or indirect transmission between herds. This was not taken into account in our study, which may explain the overestimate of vaccination duration in Jendouba and Sidi Bouzid.

Various categories of human resources were considered for vaccination tasks: official veterinarians, vaccination technicians and appointed private veterinarians. As highlighted by other studies that focused on the impact of human resources on the control of a FMD outbreak [4, 8, 12], we have shown that the feasibility of disease control strategy closely depends on the available resources. This was particularly critical considering the objective of quick completion of the emergency vaccination campaign, which could clearly neither be achieved using the standard level of public resources dedicated to FMD (scenario 1A and 1B), nor using the maximum level of public resources (scenario 2A and 2B). Increasing the human resources available, by including appointed private veterinarians (as it has been the case during the 2^nd^ phase of the vaccination campaign in 2014), appeared necessary to decrease the time necessary to vaccinate all animals. The use of appointed private veterinarians would generate an additional cost, which could probably be offset by the cost incurred in the event of an extension of the duration of the outbreak. Further studies are however needed to confirm this hypothesis.

Given the incomplete identification and lack of traceability of animals, the data on the number of herds in each governorate were based on record estimates (field interviews). The Tunisian livestock population is highly mobile, which may lead to under- or overestimate of the number of herds in the governorates, depending on the arrival or departure of animals. This may affect the duration of a vaccination campaign. In addition, the analysis considered human resources mobilised 8 hours a day, 6 days a week. In case of a FMD-crisis, it would be difficult to mobilise all available human resources identified for such a long time each week, due to the need to fulfil other tasks of the veterinary services. The resources-requirement grid was defined at the level of the herd for the tasks of vaccination. Another possibility would be to consider the number of animals that a vaccination team could vaccinate in one day, which could allow a finer estimate of the time needed to vaccinate herds, although a more accurate knowledge of the size of ruminant populations would then be necessary.

The clinical surveillance of herds in the restriction zones around FMD-infected premises is important for the management of a FMD outbreak, to detect all infected premises early and allow their rapid management to prevent the spreading of the disease. In Tunisia, the emergency plan provides for the use of state agents for such surveillance. Here, we did not consider the clinical surveillance task because it was not implemented during the 2014 FMD outbreak. However, if such surveillance had been considered, the corresponding consumption of human resources would have probably led to a lengthening of the time needed to complete the emergency vaccination. To avoid this, the clinical surveillance task could be delegated to free-practice veterinarians, with upstream consideration given to the pricing and means of payment. In the current situation of the veterinary services budget, such a possibility is not feasible. However, a project to create a Special Animal Health Fund is under discussion by the animal health authorities and the proposal could be examined. In addition, surveillance could also be improved by raising awareness among all stakeholders on the importance of the detection and reporting of the disease (veterinarians, farmers, etc.).

Included in the Tunisian contingency plan, the culling of susceptible animals in infected premises was not implemented in 2014, with some exceptions and on a voluntary basis by farmers. Given the incomplete identification of animals and ineffective movement restrictions during the earlier FMD outbreaks in Tunisia, culling appears to be essential to quickly control the spread of the virus, especially in case of the circulation of a new FMDV serotype in Tunisia different from the used vaccine. Further studies are however required (socio-economic impact, cost-benefit analysis) to assess whether culling is relevant in the Tunisian context. The lack of financial resources for the compensation of livestock lost by farmers is the main reason why slaughter is not practiced. The Special Animal Health Fund, if established, could solve this problem. Another alternative would be to introduce major outbreaks, such as FMD, into the regional Rescue Organization Plan, both to mobilise additional human resources of other institutions (potentially identified during field interviews with the question about the number of other human resources that could be mobilised for the management of a FMD outbreak) and to free up funds to compensate farmers.

The pooling of human resources can solve the possible problem of a lack of human resources for vaccination in a given governorate, such pooling could also be performed at the level of the governorate or with neighbouring governorates. Within the governorate, other Ministries could be involved in the management of the FMD outbreak. Their contribution could be to provide personnel for culling. Other institutions within the governorate could also contribute human resources, such as the municipality and the regional equipment department. This requires the upstream development of a regional emergency plan, which determines the role and commitment of each institution while increasing stakeholder awareness of the disease and of its impact. In the event of an outbreak, a governorate may be affected and not neighbouring governorates. The feasibility of deployment of human resources from the nearest governorate could be assessed (or from neighbouring countries in the context of REMESA network), although generalised vaccination limits this alternative. Such pooling should be pre-established and put in writing, with a mobilisation procedure listing the commitment of all stakeholders.

This study was carried out in the context of a changing animal health sector in Tunisia, due to discussions on the reform of veterinary services, as well as studies and projects underway to update the emergency response plan in the event of a FMD outbreak and the creation of a Special Animal Health Fund. This study could provide support to the authorities and an inventory of the human resources that could be mobilised to properly adapt and optimise the control strategy in the event of a FMD outbreak in Tunisia. In addition, the resources-requirement grid developed here could be used by the governorates to help authorities properly prepare for a potential FMD outbreak. The human resources required could, therefore, be calculated as the crisis progresses and the available human resources adjusted regularly, which would make it possible to assess and monitor the gap in human resources in times of crisis, with a view to reducing them. Finally, the resource-requirement grid could be easily adapted, extended (e.g. for the culling-related tasks) and used as a tool in other countries that would like to conduct the same type of study.

## References

[1] Knight-Jones TJ and Rushton J (2013) The economic impacts of foot and mouth disease - what are they, how big are they and where do they occur? Preventive Veterinary Medicine 112, 161–173. 23958457 10.1016/j.prevetmed.2013.07.013 PMC3989032

[2] Marcos A and Perez AM (2019) Quantitative risk assessment of foot-and-mouth disease (FMD) virus Introduction into the FMD-free zone without vaccination of Argentina through legal and illegal trade of bone-in beef and unvaccinated susceptible species. Frontiers in Veterinary Science 6, 78. 30941355 10.3389/fvets.2019.00078 PMC6433775

[3] Martinez-Lopez B (2008) Quantitative risk assessment of foot-and-mouth disease introduction into Spain via importation of live animals. Preventive Veterinary Medicine 86, 43–56. 18430478 10.1016/j.prevetmed.2008.03.003

[4] Halasa T and Boklund A (2014) The impact of resources for clinical surveillance on the control of a hypothetical foot-and-mouth disease epidemic in Denmark. PLoS One 9, e102480. 25014351 10.1371/journal.pone.0102480 PMC4094525

[5] Backer JA (2012) Vaccination against foot-and-mouth disease I: epidemiological consequences. Preventive Veterinary Medicine 107, 27–40. 22749763 10.1016/j.prevetmed.2012.05.012

[6] Brito BP (2017) Review of the global distribution of foot-and-mouth disease virus from 2007 to 2014. Transboundary and Emerging Diseases 64, 316–332. 25996568 10.1111/tbed.12373

[7] Haj A and Khorchani R (2014) Rapport de suivi de la situation sanitaire de la FA en Tunisie, Direction Générale des Services Vétérinaires Tunis.

[8] Garner MG (2016) Estimating resource requirements to staff a response to a medium to large outbreak of foot and mouth disease in Australia. Transboundary and Emerging Diseases 63, e109–e121. 24894407 10.1111/tbed.12239

[9] Davies G (2002) The foot and mouth disease (FMD) epidemic in the United Kingdom 2001. Comparative Immunology, Microbiology and Infectious Diseases 25, 331–343. 10.1016/s0147-9571(02)00030-9 12365809

[10] Bouma A (2003) The foot-and-mouth disease epidemic in The Netherlands in 2001. Preventive Veterinary Medicine 57, 155–166. 12581598 10.1016/s0167-5877(02)00217-9

[11] Direction Générale des Services Vétérinaires (2009) Plan d'intervention d'urgence contre les épizooties majeures, Direction Générale des Services Vétérinaires, Tunis

[12] Roche SE (2014) How do resources influence control measures during a simulated outbreak of foot and mouth disease in Australia? Preventive Veterinary Medicine 113, 436–446. 24412502 10.1016/j.prevetmed.2013.12.003

